# Nesfatin-1 and nesfatin-1-like peptide suppress growth hormone synthesis via the AC/PKA/CREB pathway in mammalian somatotrophs

**DOI:** 10.1038/s41598-020-73840-4

**Published:** 2020-10-07

**Authors:** Emilio J. Vélez, Suraj Unniappan

**Affiliations:** Laboratory of Integrative Neuroendocrinology, Department of Veterinary Biomedical Sciences, Western College of Veterinary Medicine, University of Saskatchewan, 52 Campus Drive, Saskatoon, SK S7N 5B4 Canada

**Keywords:** Peptide hormones, Peptides, Cell biology, Cell signalling

## Abstract

Nesfatin-1 (NESF) and NESF-like peptide (NLP), encoded in nucleobindin 2 and 1 (NUCB2 and NUCB1), respectively, are orphan ligands and metabolic factors. We hypothesized that NESF and NLP suppress growth hormone (GH) synthesis, and aimed to determine whether mammalian somatotrophs are a source and site of action of these peptides. Using immortalized rat somatotrophs (GH3 cells), NUCB expression was determined by qPCR, immunofluorescence and Western blot. NESF and NLP binding to GH3 cells was tested using fluorescence imaging. Both time- and concentration-dependent studies were performed to test whether NESF and NLP affect GH. Moreover, the ability of these peptides to modulate the effects of ghrelin, and cell-signaling pathways were studied. GH3 cells express NUCB mRNAs and protein. Labeled NESF and NLP bind to the surface of GH3 cells, and incubation with either NESF or NLP decreased GH mRNA and protein expression, downregulated *pit-1* mRNA, and blocked the GH stimulatory effects of ghrelin. Pre-incubation with either of these peptides reduced CREB phosphorylation by an AC-activator, but not when PKA was directly activated by a cAMP analog. Our results indicate that rat somatotrophs are a source of NUCBs, and that NESF and NLP downregulate GH synthesis through the AC/PKA/CREB signaling pathway.

## Introduction

In recent years, it has been observed that two DNA and calcium-binding secreted peptides named nucleobindins (NUCBs, designated as NUCB1 and NUCB2) are involved in many processes, including the activation of G protein signaling^1^. In addition, it was discovered that NUCBs could be processed by prohormone convertases giving rise to smaller bioactive peptides. The first peptide discovered was processed from NUCB2 and was called nesfatin-1 (NESF)^2^. A nesfatin-1-like peptide (NLP) composed of 77 amino acid that is processed from NUCB1 and shared a 76.6% amino acid sequence identity with NESF, has been proposed more recently in mice^3^. The physiological roles of NESF and NLP include the suppression of food intake and the modulation of energy homeostasis^2,4–8^, stimulation of insulin secretion^3,4,9^, and the control of pituitary LH and FSH^9–14^. However, the role of NLP has not been widely studied. Overall, both NESF and NLP appear to be pleiotropic hormone-like bioactive molecules.

The identity of receptors involved in NUCB-encoded peptide action remains unknown^1^. However, at least some of the functions of NESF appear to be mediated via G protein-coupled receptors (GPCRs)^15,16^. NUCBs are present within the pituitary in some species, and NESF regulates pituitary gonadotropin levels^17–20^. These results suggest that pituitary is a site of synthesis of NUCBs and its action. Although a previous study using autoradiography demonstrated NESF-binding sites in the rat pituitary^9,21^, it is still unknown whether NESF and NLP could bind to some or all types of cells present in the pituitary. One of the cell types present in the anterior pituitary is the somatotroph, which is the primary source of growth hormone (GH). Besides its clinical relevance due to the endocrine regulation of growth through the GH-insulin-like growth factor 1 (IGF-1) axis, GH is implicated in various vital processes in vertebrates, including nutrition, metabolism, reproduction, physical activity, neuroprotection and immunity^22–27^. The hypothalamus mainly regulates GH levels through a stimulator, GH-releasing hormone (GHRH), and an inhibitor, somatostatin^22–24,28^. Other factors, including ghrelin (GRL), IGF-1, or even the levels of GH itself, determine GH release^24,25^. At the cellular level, the control of the GH in somatotrophs is triggered through the modulation of GPCRs^25,29^. The activation of stimulatory Gα-subunits (Gαs) increases the activity of adenylyl cyclase to produce cAMP and modulates the Ca^2+^-channels to facilitate GH release^30^. The rise in cAMP activates protein kinase A (PKA), which phosphorylates key proteins, including the transcription factor cAMP-responsive element-binding protein CREB at serine 133. The phosphorylated CREB (P-CREB) stimulates the expression of different genes, including the pituitary-specific positive transcription factor 1 (*pit-1*), which in turn stimulates the expression of *gh*^24,29,31^. In contrast, the activation of inhibitory Gα-subunits (Gαi), as it happens with somatostatin, blocks both Ca^2+^-channels and adenylyl cyclase activity that results in a reduction in GH^29,30^.

We hypothesized that NUCB-encoded peptides, NESF and NLP, suppress GH synthesis in somatotrophs. This main objective of this research was to determine whether mammalian somatotrophs are a source and site of action of NESF and NLP. An in vitro model (GH3 and RC-4B/C cell lines) was employed to test if NESF and NLP regulate GH gene and protein expression and determine the main signaling pathways (GH3 cells) mediating their action on somatotrophs.

## Results

### Somatotrophs express NUCBs and both NESF and NLP bind to GH3 cell surface

**Figure 1:**
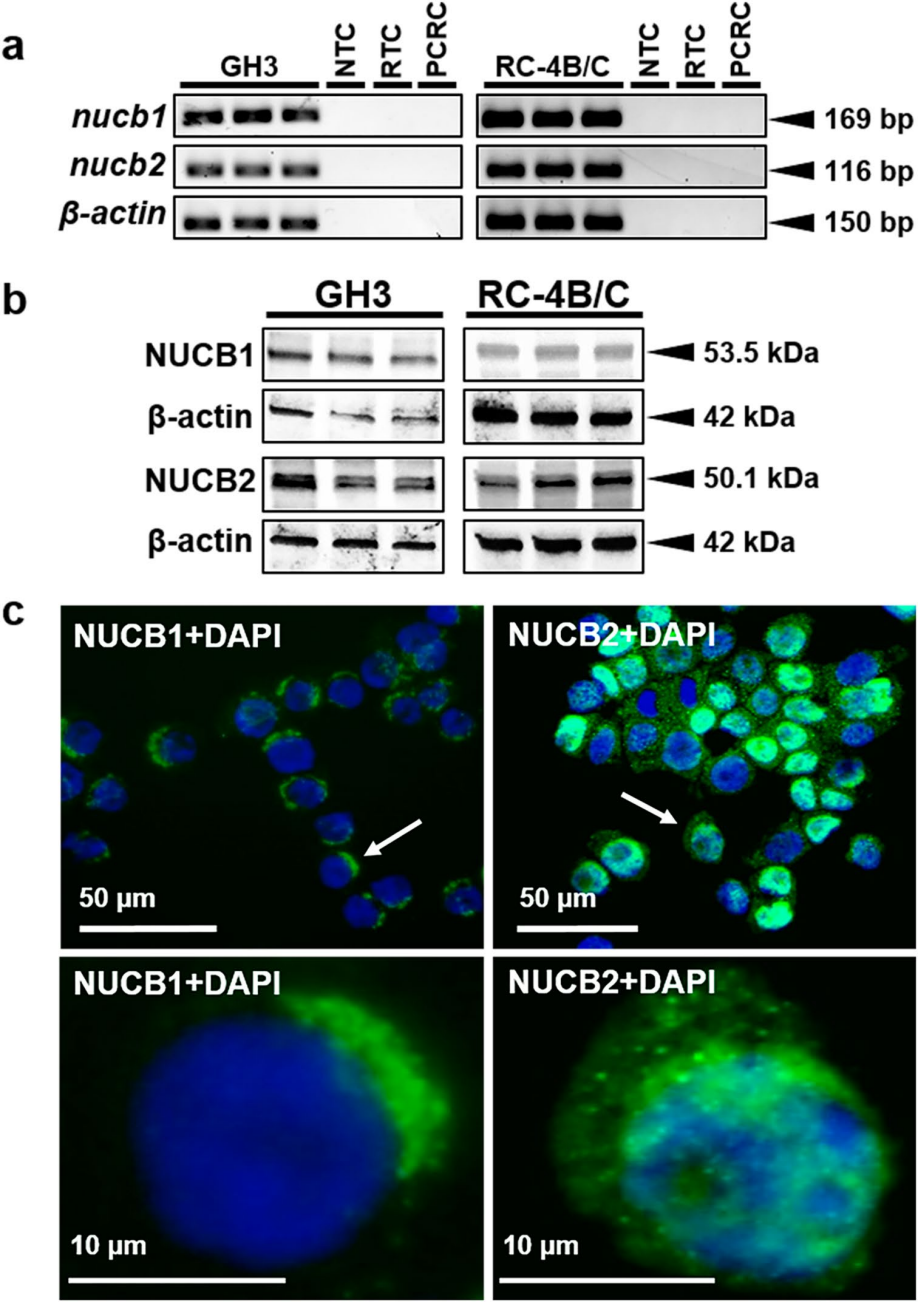
Mammalian somatotrophs express both NUCB1 and NUCB2. (**a**) Representative gel images showing *nucb1*, *nucb2* and *β-actin* PCR products from both GH3 and RC-4B/C rat somatotroph cells. NTC: No Template Control. RTC: No Reverse Transcriptase Control. PCRC: PCR Control (No Template Control in the PCR). (**b**) Representative immunoreactive bands of NUCB1, NUCB2 and β-actin analyzed by Western blot in protein extracts from either GH3 or RC-4B/C rat somatotroph cells. (**c**) Representative images of NUCB1 and NUCB2 (in green) protein expression in GH3 cells counterstained with DAPI (in blue) and detected by immunofluorescence. The images below correspond to an amplification of the area indicated with an arrow in the corresponding image above. Images were acquired at 40X magnification.

**Figure 2:**
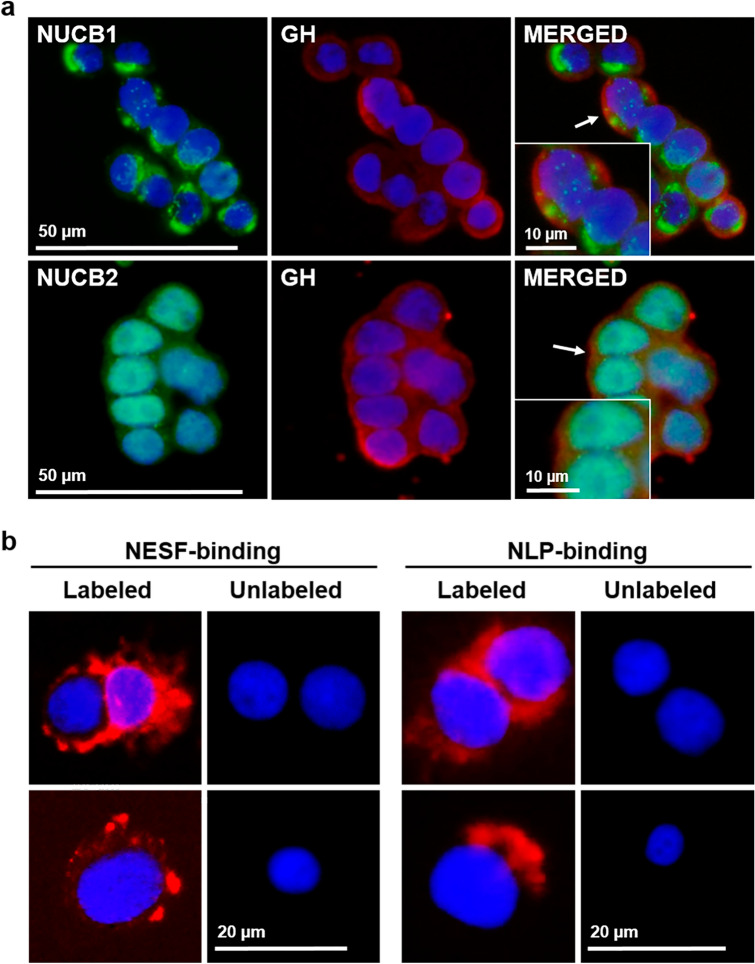
(**a**) NUCB1/NLP and NUCB2/NESF colocalizes with GH in somatotrophs. Representative images of immunofluorescence detection of NUCB1 (green), NUCB2 (green) and GH (red) in GH3 cells. Cells were counterstained with DAPI (blue) and the images were acquired at 40X magnification. Representative and magnified images corresponding to the areas indicated with an arrow in the merged figure are shown in the inset. **(b)** NESF and NLP bind to mammalian somatotroph cells. Representative images of NESF-binding (left) and NLP-binding (right) detection (in red) in the surface of GH3 cells after incubation for 1 h with 1 nM CF568-labeled NESF or NLP, or unlabeled-peptides, followed by several washes with PBS. GH3 cells were counterstained with DAPI (in blue), and images were acquired at 40X magnification.

*Nucb1* and *nucb2* mRNA expression was detected in both GH3 and RC-4B/C cells (Fig. 1a; see Supplementary Fig. S1 online). Similarly, NUCB1 and NUCB2 protein were found in both cell lines used (Fig. 1b). The bands observed by WB analyses corresponded to the expected size for NUCB1 (53.5 kDa) and NUCB2 (50.1 kDa). NUCB1/NLP immunoreactivity was mainly located in the cytoplasm of GH3 cells (Fig. 1c). In contrast, NUCB2/NESF showed a more diffuse distribution, and was also present in the nucleus. GH3 cells stained positive for GH within the cytoplasm were also immunopositive for both NUCB1 and NUCB2 (Fig. 2a), and similar results were observed with RC-4B/C cells (see Supplementary Fig. S2 online). The ligand-binding assay showed that both CF568-labeled-NESF and CF568-labeled-NLP bind to the membrane of GH3 cells (Fig. 2b), suggesting a possible GPCR-mediated action of NESF and NLP in these cells.

### NESF and NLP suppress GH mRNA and protein expression in somatotroph cells

**Figure 3:**
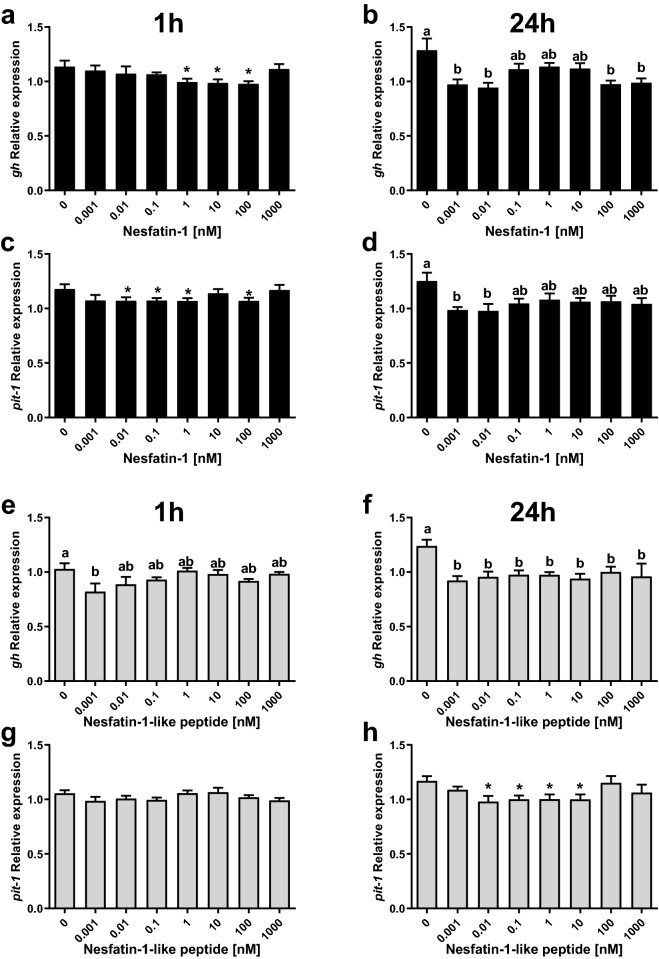
The incubation with either NESF or NLP at different concentrations and times decreases the gene expression of *gh* and the transcription factor *pit-1* in somatotrophs. Effects of 1 h (left column) or 24 h (right column) incubation with NESF (**a**–**d**) or NLP (**e**–**h**) on the gene expression of *gh* (**a**,**b**,**e**, and **f**) and *pit-1* (**c**,**d**,**g**, and **h**) in GH3 cells. Four independent experiments with triplicates (n = 3 wells/treatment/experiment) were performed for each study. Data from all four experiments were pooled to conduct statistical analyses and are shown as mean ± SEM (n = 12 wells) relative to the reference genes *β-actin* and *rpl13*. Different letters indicate significant differences (*p* < *0.05*) between the different concentrations detected by one-way ANOVA test followed by Tukey’s multiple comparison test. When no differences were found with Tukey’s test, Student’s t-test was used to detect differences with the Control (0 nM), and are denoted with asterisks (*p* < *0.05*).

**Figure 4:**
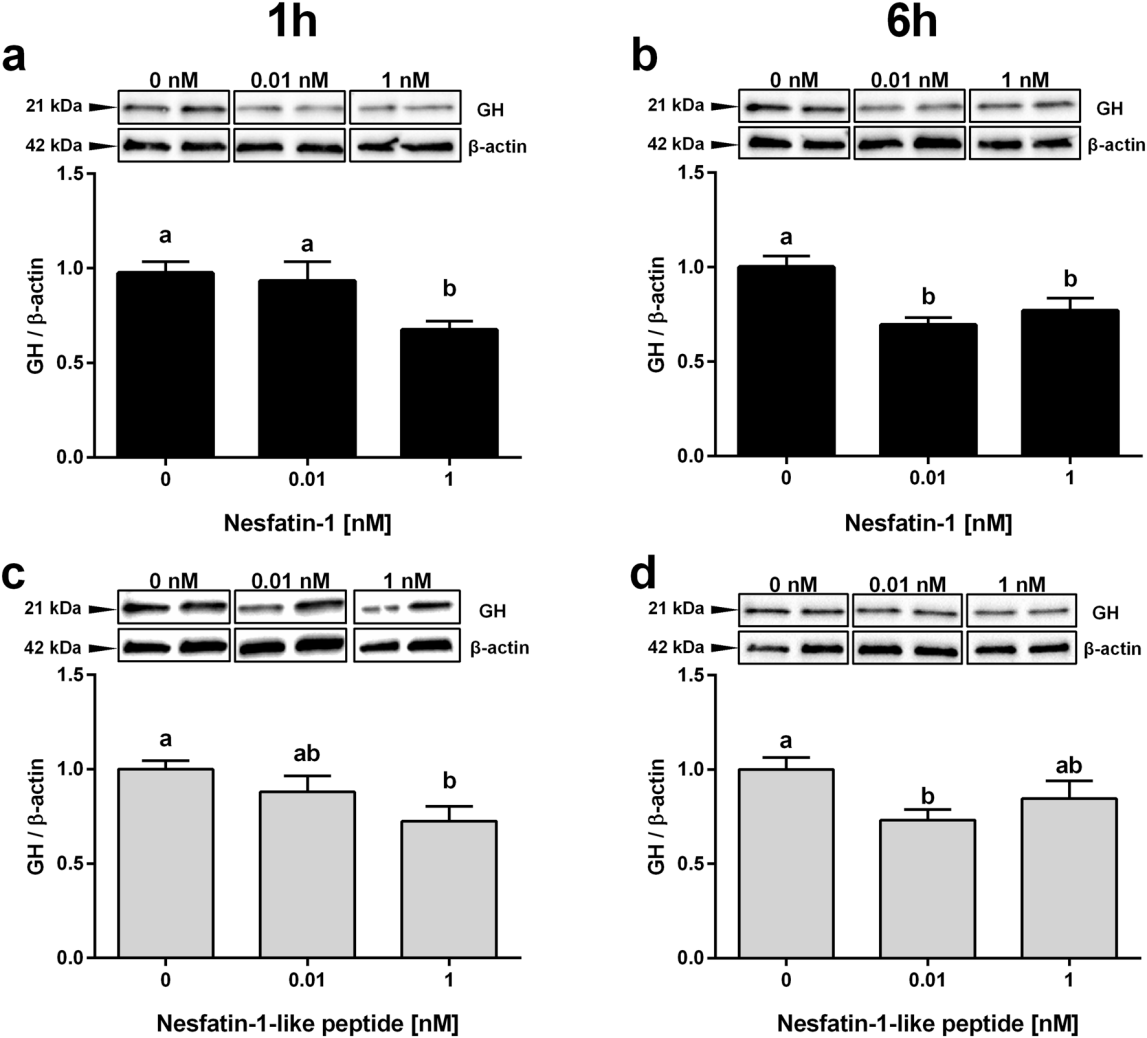
NESF or NLP treatment decreases GH protein in mammalian somatotroph cells. Effects of 1 h incubation with NESF (**a**) or NLP (**c**), or of 6 h incubation (**b** and **d**, respectively), on the GH protein levels in GH3 cells detected by Western blot. Representative immunoreactive bands and quantification of GH band intensity. Four independent experiments with triplicates (n = 3 wells/treatment/experiment) were performed for each study. Data from all four experiments were pooled to conduct statistical analyses and are shown as mean ± SEM (n = 12 wells) normalized to the levels of β-actin and presented as a fold change over Control. Different letters indicate significant differences (*p* < *0.05*) between the different concentrations detected by one-way ANOVA test followed by Tukey’s multiple comparison test.

**Figure 5:**
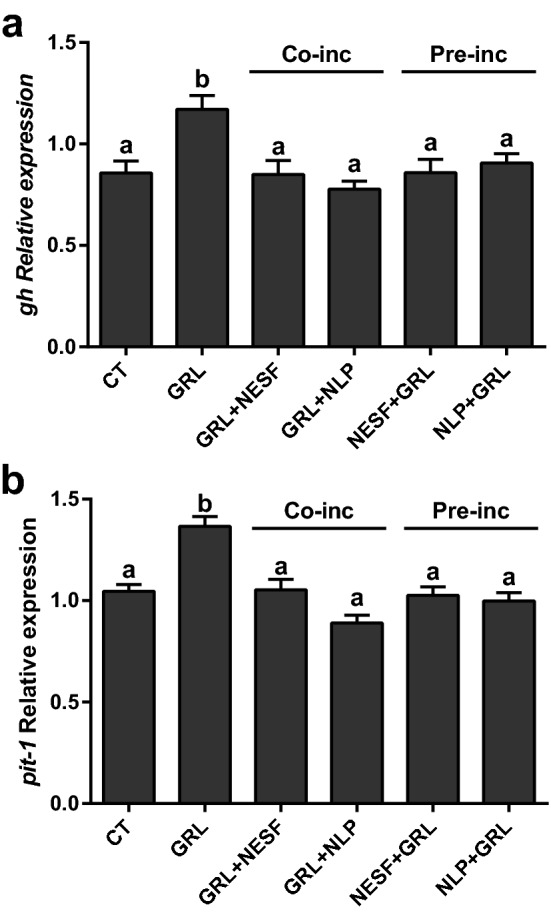
The co-incubation or pre-incubation with either NESF or NLP block the stimulatory effects of GRL on both *gh* and *pit-1* mRNA levels in somatotrophs. Effects of pre-incubation for 150 min with growth culture media, or either 1 nM NESF or 1 nM NLP (labeled Pre-inc), or 10 nM GRL in combination with 1 nM NESF or NLP (as Co-inc) for 2 h, on the regulation of *gh* (**a**) and *pit-1* (**b**) gene expression. For the “pre-incubation”, cells were either incubated with growth culture media alone, or media supplemented with 1 nM NESF or 1 nM NLP for 150 min. Then, cells were again incubated for 120 min with fresh media alone (CT), or media supplemented with 1 nM NESF + 10 nM rodent GRL, or 1 nM NLP + 10 nM rodent GRL^40^. Cells were then washed twice with 1X PBS before the collection of total RNA, as explained below. In the “co-incubation” study, cells were either treated with plain growth media (CT), or with 1 nM NESF + 10 nM rodent GRL or 1 nM NLP + 10 nM rodent GRL for 120 min (no pre-treatment with nesfatin-1 or NLP in this co-incubation study). Four independent experiments with triplicates (n = 3 wells/treatment/experiment) were performed for each study. Data from all four experiments were pooled to conduct statistical analyses and are shown as mean ± SEM (n = 12 wells) relative to the reference genes *β-actin* and *rpl13*. Different letters indicate significant differences (*p* < *0.05*) between the different conditions detected by one-way ANOVA test followed by Tukey’s multiple comparison test.

Incubation with 1, 10, and 100 nM of NESF for 1 h reduced *gh* mRNA expression in GH3 cells (Fig. 3a). The highest reduction (13.7%) was caused by 100 nM of NESF. NLP (0.001 nM) also downregulated *gh* expression at 1 h (Fig. 3e), in this case a 20.2%. Both peptides also significantly reduced the expression of *gh* at 24 h incubation (Fig. 3b,f), although with slight differences. More specifically, both low (0.001 and 0.01 nM), and high (100 and 1000 nM) concentrations of NESF were effective in reducing the expression of *gh* at 24 h (maximum reduction of 26.7% with 0.01 nM NESF), but this was not observed with medium concentrations (which induced a non-significant average reduction of 13.7%). In the case of NLP, all concentrations tested (from 0.001 to 1000 nM) significantly reduced *gh* mRNA levels in an average of 22.8% at 24 h, with a maximum reduction of 25.6% observed at 0.001 nM. Besides, the expression of the pituitary-specific positive transcription factor 1 (*pit-1*) was significantly downregulated by NESF at 1 (up to 9.5%) and 24 h (21.8% at 0.01 nM) (Fig. 3c,d). Although 1 h incubation with NLP only induced a slight (6.6% at 0.001 nM), non-significant reduction in the expression of *pit-1* (Fig. 3g), 24 h treatment with 0.01 to 10 nM NLP significantly reduced *pit-1* mRNA levels (Fig. 3h; a maximum reduction of 16.3% by 0.01 nM of NLP). NESF (1 nM) reduced in a 30.8% the GH protein levels at 1 h (Fig. 4a), whereas 0.01 nM was effective in causing a 30.5% reduction in GH protein after 6 h treatment (Fig. 4b). Meanwhile, similar suppressive effects were observed for NLP at both 1 (27.5%) and 6 h (26.9%) incubation (Fig. 4c,d). Likewise, incubation for 1 h with NESF at both 0.001 and 0.1 nM significantly reduced (about 50%) the expression of *gh* in RC-4B/C cells (see Supplementary Fig. S2 online). On the other hand, the incubation of GH3 cells with 10 nM ghrelin (GRL) significantly increased the gene expression of *gh* (Fig. 5a) and *pit-1* (Fig. 5b), and both co-incubation and pre-incubation with either 1 nM NESF or NLP blocked the GRL effects and recovered the expression of the control group.

### Mechanism of action

**Figure 6:**
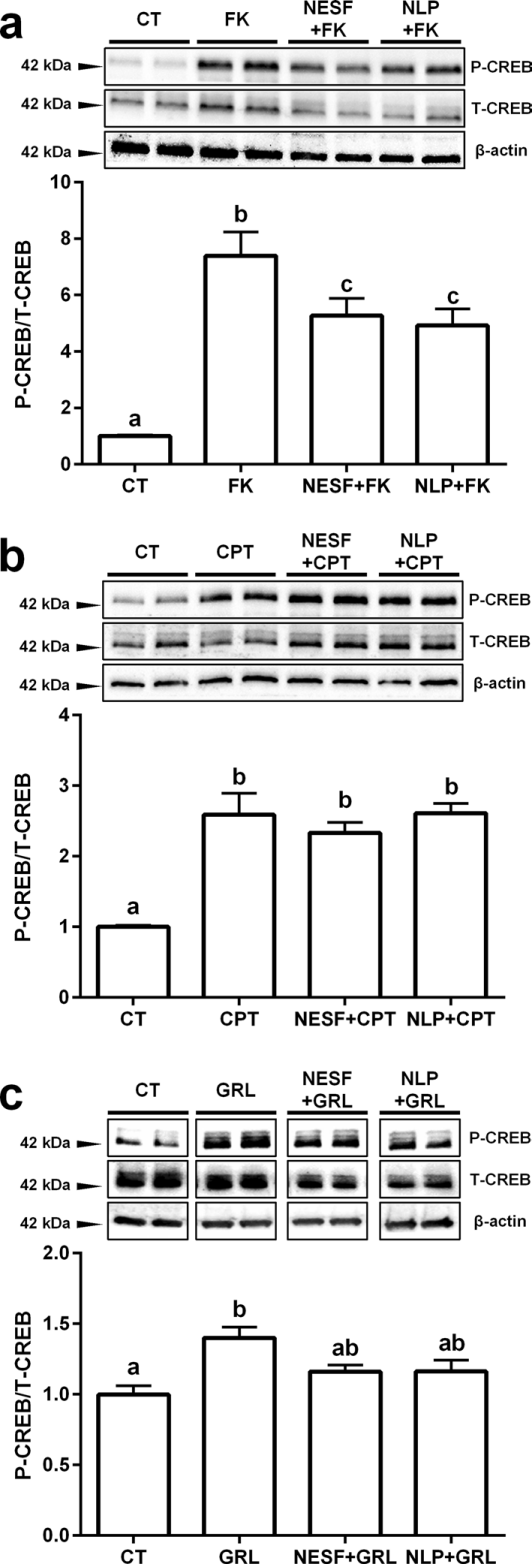
The mechanism of action of both NESF and NLP in the somatotrophs involves the modulation of the cAMP/PKA/CREB signalling pathway. The effects of NESF or NLP pre-incubation on the CREB phosphorylation ratio by forskolin (FK) (**a**), CPT (**b**), or GRL (**c**) were evaluated. Representative immunoreactive bands of phosphorylated CREB (P-CREB), total CREB (T-CREB) and β-actin, and quantification of band intensity of P-CREB normalized to T-CREB. Four independent experiments with triplicates (n = 3 wells/treatment/experiment) were performed for each study. Data from all four experiments were pooled to conduct statistical analyses and are shown as mean ± SEM (n = 12 wells) and presented as a fold change over Control. Different letters indicate significant differences (*p* < *0.05*) between the different treatments detected by one-way ANOVA test followed by Tukey’s multiple comparison test. When two groups share one letter, they are not statistically different.

When GH3 cells were pre-incubated with either 1 nM NESF or NLP, the activation of the PKA/CREB signaling pathway by the classical activator of adenylyl cyclase (AC), forskolin (FK) was significantly decreased, as indicated by the diminished (31% on average) phosphorylation ratio of CREB (Fig. 6a). In contrast to what was found with FK, neither NESF nor NLP reduced the phosphorylation of CREB by the cAMP analog CPT (Fig. 6b). On the other hand, pre-incubation with either NESF or NLP also prevented the stimulatory effects of the GH secretagogue GRL on the analyzed signaling pathway (Fig. 6c).

## Discussion

The search for novel regulators of somatotrophs could open new avenues to manage growth disorders. With this in mind, we aimed to analyze the role of recently discovered NESF and NLP on GH synthesis in mammals using an in vitro model. The results obtained revealed that rat somatotrophs express NUCB1 and NUCB2 mRNAs and protein. These results are in agreement with previous observations of NUCB1 and NUCB2 in the anterior pituitary of rodents^17–19^. However, in the anterior region of goldfish pituitary, while NUCB1/NLP was found^8^, NUCB2/NESF was not observed^12^. In the present study, NUCB1/NLP immunoreactivity in GH3 cells appeared mainly in the cytoplasm. In previous studies in rats and yeast, NUCB1 was found in the Golgi apparatus, probably associated with the control of Ca^2+^ during cell signaling events^32,33^. NUCB2/NESF immunoreactivity showed a more diffuse presence compared to NUCB1/NLP, and it was detected in the nucleus. Nuclear staining of both peptides in the rat pituitary gland was previously reported^19^. Future studies (e.g. subcellular fractionation) are required to determine the organelle-specific localization of NUCBs in the somatotrophs. However, the observed expression of both NUCB1 and NUCB2 suggests that nucleobindins and encoded peptides could play a role in regulating somatotrophs. In support of this, NESF-binding sites were found in the rat pituitary^9,21^. Here, our fluorescent-labeled ligand-assay found that both NESF and NLP bind to the surface of GH3, providing additional support for the direct action of these two peptides on somatotrophs.

Next, we tested whether NESF and NLP indeed could act directly on rat somatotrophs. The incubation of somatotrophs with NESF or NLP significantly decreased *gh* mRNA expression and GH protein levels, although time- and concentration-dependent effects were observed. While 0.001 nM of NLP induced a maximum reduction of *gh* mRNA at 1 h incubation (20.2%), the maximum effect of NESF (13.7%) was caused by higher concentration (100 nM). At 24 h, whereas all the concentrations of NLP reduced *gh* gene expression, only low and high concentrations of NESF were effective. These results might suggest that NLP could exert stronger or faster modulation of the *gh* transcription than NESF, or even that different receptors could modulate each peptide´s effects. It is possible that multiple receptors mediate the effects of NESF on GH. It is possible that one receptor mediates the effects at lower doses, and these effects are saturated at the medium doses tested. Meanwhile, a second set of receptors becomes functional/activated at the higher concentration of NESF. In this sense, and in contrast to that observed in *gh* mRNA, the expression of *pit-1* was also significantly downregulated by both peptides at 24 h, but only by NESF at 1 h. In addition to these different time-effects, the degree of reduction on *pit-1* mRNA was somewhat higher in the case of NESF (21.8%) than of NLP (16.3%). These results could reinforce the idea that, beyond different potency, NESF and NLP could act through different receptors in the somatotrophs. Nevertheless, as the presence of Pit-1 is critical for the expression of *gh*, it is expected that even a slight reduction in *pit-1*, such as the 6.6% decline observed with NLP at 1 h, could be translated to a higher decrease in *gh* mRNA. In fact, the reduction degrees observed on the GH protein levels were comparable among peptides and incubation times, ranging from 26.9% to 30.8% relative to the control group.

**Figure 7:**
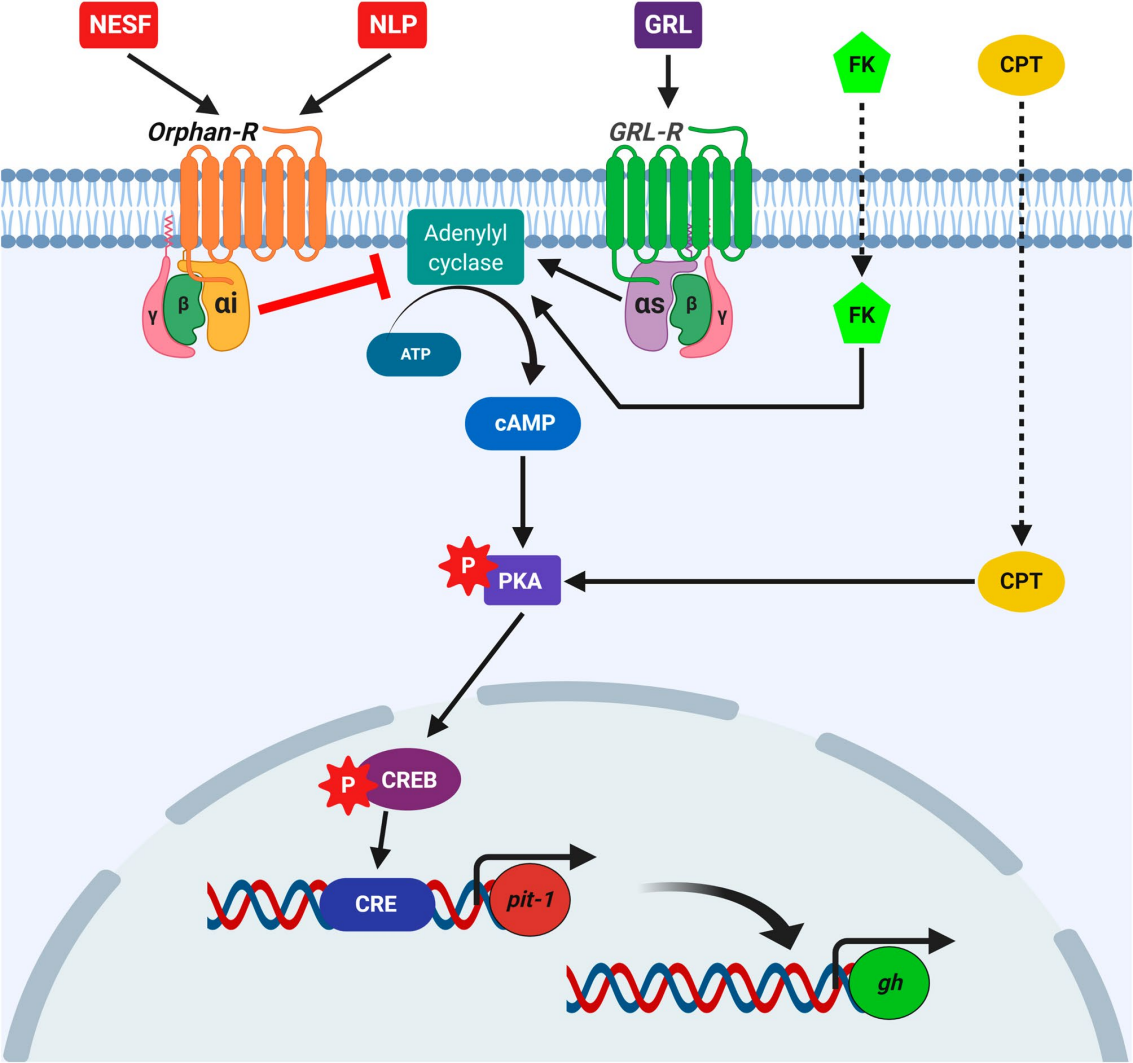
Schematic representation of the proposed putative mechanism of action of NESF and NLP in the regulation of GH synthesis in the mammalian somatotrophs. While the GH-secretagogue ghrelin (GRL) activates the cAMP/PKA/CREB pathway to stimulate the synthesis of GH through a GPCR with a Gα-stimulatory subunit (Gαs), NESF and NLP prevent the activation of this signalling pathway by the adenylyl cyclase stimulator forskolin (FK). However, the phosphorylation of CREB is not compromised when the pathway is directly activated at the level of PKA by the cAMP-analog CPT. These results suggest that exogenous nucleobindin encoded peptides likely exert their effects by the inhibition of the adenylyl cyclase, possibly through a GPCR associated with a Gα-inhibitory subunit (Gαi). Figure created with BioRender.com tools.

We also found that the co-incubation or pre-incubation with either NESF or NLP blocked the stimulatory effects of ghrelin on *gh* and *pit-1* mRNA levels. Therefore, although the possibility that NESF and NLP exert their effects through different receptors cannot be ruled out, the functions of both peptides seem to be highly conserved. Together, these results demonstrated that NESF and NLP act directly on rat somatotrophs to regulate both basal and GRL-induced GH expression. Thus, GH regulation is a newly identified function of NESF and NLP. Future experiments are necessary to better understand the mechanism of action and the dynamics of the receptors mediating the effects of NESF and NLP. In this sense, although it is expected that nucleobindin encoded peptides actions are mediated by GPCRs^1,4,15,16^, the identity of the receptors is still unknown. In this research, we then tested the main synthetic signaling pathways in somatotrophs (i.e. the cAMP/PKA/CREB) to gather more data about the GPCRs that might be involved in NESF and NLP actions on somatotrophs. Pre-incubation with either of those peptides significantly reduced by 31% the phosphorylation of CREB by FK. This result suggests that nucleobindin-encoded peptides influence the AC activity in GH3 cells. This fact could prevent a rise in cAMP that affects the downstream signaling, which consequently will affect GH production. Besides, we found that CREB phosphorylation by the direct activation upstream of the signaling pathway (at the PKA-level by the cAMP-analog CPT), was not altered by the pre-incubation with either NESF or NLP. This result suggests that the effect of NESF and NLP in downregulating the cAMP/PKA/CREB signaling pathway is compensated with the addition of CPT, supporting that NESF and NLP act at the AC level. In addition, pre-incubation with either NESF or NLP also blocked the stimulatory effects of ghrelin on CREB phosphorylation, further supporting that the inhibition of the mentioned signaling pathway is part of the mechanism of action of both NESF and NLP to modulate both basal and GRL-induced regulation of GH. Overall, these results demonstrated that nucleobindin-encoded peptides modulate basal and GRL-induced GH synthesis in mammalian somatotrophs by a mechanism that likely involves the inhibition of AC. This suggests that in somatotrophs, NESF and NLP actions could be mediated by a GPCR associated with a Gα-inhibitory subunit (Gαi) (Fig. 7), as it occurs with other GH inhibitors^34–36^. Although this study shows the involvement of the cAMP-AC pathway mediating the effects of NESF and NLP on GH, other mediators cannot be excluded and should be pursued in future.

In summary, the present study demonstrates that rat somatotrophs are a source of NUCBs and their encoded peptides. Our results indicate that both NESF and NLP act directly on somatotrophs to downregulate the synthesis of GH acting via a GPCR. The proposed putative signaling mechanism that likely includes a Gαi subunit is shown in Fig. 7. Overall, the present work identifies a new physiological function for nucleobindin-encoded peptides and provides new information that can contribute to the identification of the putative GPCR involved. Future research on the role of endogenous NUCBs and encoded peptides, its mechanism of action and effects on GH secretion warrant consideration. In conclusion, we present a new function for NESF and NLP and information that begins a better understanding of cell signaling mediated by both peptides.

## Methods

### Cell culture and immunocytochemistry

The GH3 cells (RRID: CVCL_0273, cat no. CCL-82.1, ATCC, USA) and RC-4B/C cells (RRID: CVCL_3785, cat no. CRL-1903, ATCC) were grown at 37 °C and 5% CO_2_ with the corresponding complete growth medium following the supplier’s recommendations. All experiments were performed using cells under passage 10, and 2.5 × 10^5^ cells/mL were plated onto chamber slides (cat no. 177437, THERMO FISHER SCIENTIFIC, USA) for immunolocalization and binding studies, and in 24-well plates (cat no. 662 160, GREINER BIO-ONE, Austria) for RNA extraction, or 12-well plates (cat no. 665 180, GREINER BIO-ONE) for protein collection.

The immunolocalization of GH, NUCB1 and NUCB2 was performed using the protocol previously described^37^ with minor modifications. Cells were blocked with an antibody blocking buffer (ABB) based in PBS consisting of 3% BSA, 0.05% Triton X-100 and 10% of protein block solution (cat no. ab64226, ABCAM, UK), and the primary and secondary antibodies were diluted in a commercial solution (cat no. ab64211, ABCAM). Finally, the preparations were mounted using VECTASHIELD mounting medium with DAPI (cat no. H1200, VECTOR LABORATORIES, USA). Cells were analyzed under a BX51 microscope (OLYMPUS, Canada), and the images were captured using an OLYMPUS DP70 camera. The primary antibodies used were mouse monoclonal to GH (1:100 dilution; RRID: AB_10547918, cat no. CLX130AP, CEDARLANE, USA), rabbit anti-mouse NUCB1 (1:200; custom synthesized, cat no. 1312-PAC-02, PACIFIC IMMUNOLOGY, USA) and rabbit anti-mouse NUCB2 (1:200; custom synthesized, cat no. 1312-PAC-01, PACIFIC IMMUNOLOGY). The antibodies used to detect NUCB1 and NUCB2 distinguishes both the precursor NUCB1 and processed NLP, and NUCB2 and NESF, respectively, but does not cross-react with the related peptide. These antibodies have been previously validated by our research group^8,20^. Besides, no-primary antibody-negative controls were included (see Supplementary Fig. S3 online). The secondary antibodies used were goat anti-mouse Alexa Fluor 594 (1:200; RRID: AB_2534091, cat no. A-11032, THERMO FISHER SCIENTIFIC), and goat anti-rabbit Alexa Fluor 488 (1:200; RRID: AB_2576217, cat no. A-11034, THERMOFISHER SCIENTIFIC).

### NESF and NLP binding

To test the ability of NESF and NLP to bind to somatotrophs, 1 pmol of either rat NESF or NLP was labeled to a fluorescent dye using the reagents of the Mix-n-Stain CF568 Small Ligand Labeling Kit (cat no. 92351, BIOTIUM INC., USA) following the manufacturer’s protocols. GH3 cells were incubated with 1 nM of NESF- or NLP-labeled, or with non-labeled peptides (negative controls) for 1 h. Then, the media was removed, and cells were washed thrice with 1X PBS to remove the unbound-peptides. Finally, the preparations were mounted and subsequently imaged using fluorescence microscopy as stated above.

### In vitro experiments

#### GH regulation

For testing the effects of NESF or NLP on GH, cells at 90% of confluence were used. The growth culture media was supplemented with rat NESF (custom synthesized peptide^6^, > 95% purity, ABGENT, USA) or rat NLP (custom synthesized peptide^7^, > 95% purity, ABGENT) at 0 (Control), 0.001, 0.01, 0.1, 1, 10, 100 or 1000 nM concentration and cells were incubated for 1, 6 or 24 h. The 24 h time point was chosen as previous studies with the same cell lines reported mRNA changes at that time ^38,39^. After incubation, cells were washed twice with 1X PBS before the collection of total RNA or the protein content, as explained below. Two separate experiments were conducted to study whether co-incubation or pre-incubation with NESF or NLP modulates the effects of ghrelin (GRL) on *gh* and *pit-1* gene expression. For the “pre-incubation”, cells were either incubated with growth culture media alone, or media supplemented with 1 nM NESF or 1 nM NLP for 150 min. Then, cells were again incubated for 120 min with fresh media alone (CT), or media supplemented with 1 nM NESF + 10 nM rodent GRL (cat no. 031-31 PHOENIX PHARMACEUTICALS, USA), or 1 nM NLP + 10 nM rodent GRL^40^. Cells were then washed twice with 1X PBS before the collection of total RNA, as explained below. In the “co-incubation” study, cells were either treated with plain growth media (CT), or with 1 nM NESF + 10 nM rodent GRL or 1 nM NLP + 10 nM rodent GRL for 120 min (no pre-treatment with NESF or NLP in this co-incubation study). Four independent experiments with triplicates (n = 3 wells/treatment/experiment) were performed for each study. Data from all four experiments were pooled to conduct statistical analyses.

#### Mechanism of action

In the cell signaling study, GH3 cells were pre-incubated for 150 min with control media (CT), or media containing 1 nM NESF or 1 nM NLP. Following this, cells were incubated for 30 min with fresh media alone, media supplemented with 1 nM NESF or 1 nM NLP (as corresponding), in combination with the cell-permeable activator of AC [FK, cat no. B1421 APEXBIO TECH., USA, at 10 µM^41^], with the lipophilic activator of cyclic AMP-dependent PKA the cAMP-analog 8-CPT-cyclic AMP (sodium salt) [CPT, cat no. 12011, CAYMAN CHEMICAL, USA, at 40 µM^42^], or with 10 nM rodent GRL^40^. After incubation, cell protein content was collected and stored as explained elsewhere. Four independent experiments with triplicates (n = 3 wells/treatment/experiment) were performed for each study. Data from all four experiments were pooled to conduct statistical analyses.

#### mRNA expression

Total RNA was extracted using 0.5 mL of RiboZol reagent (cat no. N580, VWR, USA), following the manufacturer’s instructions. Then, RNA concentration and purity were determined using a NanoDrop2000 (THERMO FISHER SCIENTIFIC). 1 µg of RNA was reverse transcribed into cDNA using the iScript Reverse Transcription Supermix for RT-qPCR (cat no. 170884 BIO-RAD, Canada). The mRNA transcripts levels were measured by quantitative real-time PCR (qPCR) in a CFX Connect Optic module (BIO-RAD) following the requirements of the MIQE guidelines^43^. The analyses were performed in triplicate in a final volume of 10 µL, including 5 µL of SensiFAST SYBR No-ROX MIX (cat no. BIO-98050, BIOLINE, UK), 500 nM of forward and reverse primers (see Supplementary Table S1 online), and 1 µL of cDNA for each corresponding sample. Prior to the analyses, a dilution curve with a pool of samples was run to confirm the specificity of the reaction and the absence of primer-dimers, as well as to determine the appropriate cDNA dilution for each assay. The mRNA levels of each gene were calculated following the Pfaffl method^44^ relative to the geometric mean of the two more stable housekeeping genes using the CFX Manager 3.1 software (BIO-RAD). To study the gene expression of *nucb1*, *nucb2* and *β-actin* in GH3 and RC-4B/C cells, each PCR product was separated by 1.5% agarose gel electrophoresis and visualized using RED Safe Nucleic Acid Staining Solution (cat no. 21141, FROGGABIO, Canada) in a ChemiDoc MP Imaging System (BIO-RAD). Full-length agarose gels are presented in Supplementary Gels online.

#### Protein expression

Protein homogenates (20 µg) were electrophoresed as previously described^37^ with minor modifications. SDS-PAGE were performed on 8–16% Mini-Protean TGX gels (cat no. 456-1104, BIO-RAD) and proteins were transferred to nitrocellulose membranes (cat no. 1704158, Bio-Rad) using the Trans-Blot Turbo Transfer System (BIO-RAD). 1X RapidBlock Solution (cat no. M325, VWR) was used for blocking the membranes and for diluting all the antibodies. The primary antibodies used were rabbit anti-mouse NUCB1 (1:2000; cat no. 1312-PAC-02) or anti-mouse NUCB2 (1:2000; cat no. 1312-PAC-01), polyclonal goat anti-GH (1:500; RRID: AB_354573, cat no. AF1067, R&D SYSTEMS, USA), monoclonal mouse anti-Actin (1:1000; RRID: AB_528068, cat no. JLA20, DEVELOPMENTAL STUDIES HYBRIDOMA BANK, University of Iowa, USA), monoclonal rabbit anti-Phospho-(Ser133)-CREB (1:1000; RRID: AB_2561044, cat no. 9198, CELL SIGNALING, USA) and monoclonal rabbit anti-CREB (1:1000; RRID: AB_331277, cat no. 9197, CELL SIGNALING). The secondary antibodies [rabbit anti-goat, goat anti-mouse and goat anti-rabbit] IgG-HRP conjugated antibodies (RRID: AB_11125144, AB_11125547, AB_11125142, cat numbers 172-1034, 170-6516 and 170-6515, respectively; BIO-RAD) were used at 1:5000 dilution. Finally, the different immunoreactive bands were developed using the Clarity Western ECL Substrate (cat no. 170-5061, BIO-RAD), and the images were captured using the ChemiDoc MP Imaging System (BIO-RAD). The bands were quantified by densitometry using ImageJ (NATIONAL INSTITUTES OF HEALTH, Bethesda, MD, USA). Full-length blots/gels are presented in Supplementary Blots online.

### Statistical analyses

Data were analyzed using IBM SPSS Statistics v.22 and are presented as mean ± SEM. Initially, normal distribution and homogeneity of variances were tested by a Shapiro–Wilk test, followed by Levene’s test. Secondly, a one-way ANOVA followed by Tukey’s multiple comparison test was used to evaluate the differences between the different experimental groups (i.e. concentrations tested). In addition, Student’s t-test was employed to compare between control (0 nM) and each concentration of peptide tested when no differences were found with Tukey’s test. Statistical differences were considered at *p* < 0.05.

## Supplementary information

Supplementary Information.

## Data Availability

The data of this study are available from the corresponding author upon reasonable request.
