# Supplementary material for: Nesfatin-1 and nesfatin-1-like peptide suppress growth hormone synthesis via the AC/PKA/CREB pathway in mammalian somatotrophs
Source: Sci Rep. 2020 Oct 7;10:16686. doi: 10.1038/s41598-020-73840-4 (PMC7541516; doi:10.1038/s41598-020-73840-4)

Nesfatin-1 and nesfatin-1-like peptide suppress growth hormone synthesis via the AC/PKA/CREB pathway in mammalian somatotrophs

Emilio J. Vélez, Suraj Unniappan*

Laboratory of Integrative Neuroendocrinology, Department of Veterinary Biomedical Sciences, Western College of Veterinary Medicine, University of Saskatchewan, Saskatoon, Canada

Supplementary Material

- **Fig. S1.** mRNA levels of *nucb1* and *nucb2*
- **Fig. S2. (a)** Colocalization of NUCB1 and NUCB2 with GH and **(b)** effects of nesfatin-1 (NESF) incubation on *gh* gene expression in RC-4B/C cells.
- **Fig. S3.** No-primary antibody-negative controls
- **Table S1.** Primer sequences.
- **Gels**: Agarose gels used in **Figure 1a**.
- **Blots:** Western blot gels used in **Figures 1**, **4** and **6**.

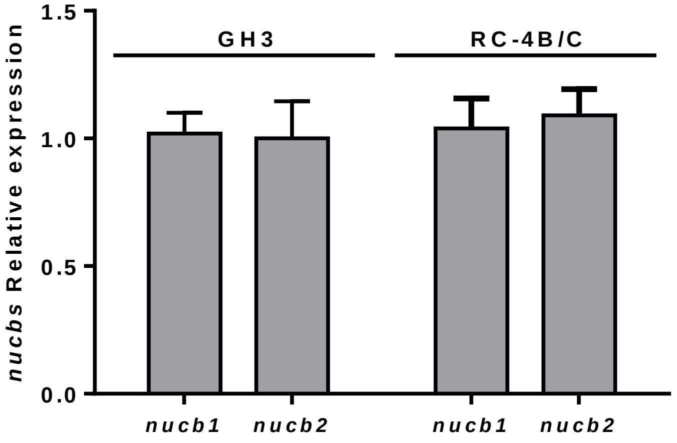

Fig. S1. Mammalian somatotrophs express both NUCB1 and NUCB2. Representative mRNA levels of *nucb1* and *nucb2* in both GH3 and RC-4B/C rat somatotroph cells. Data are shown as mean ± SEM (n = 3 wells) relative to the reference genes *β-actin* and *rpl13*.

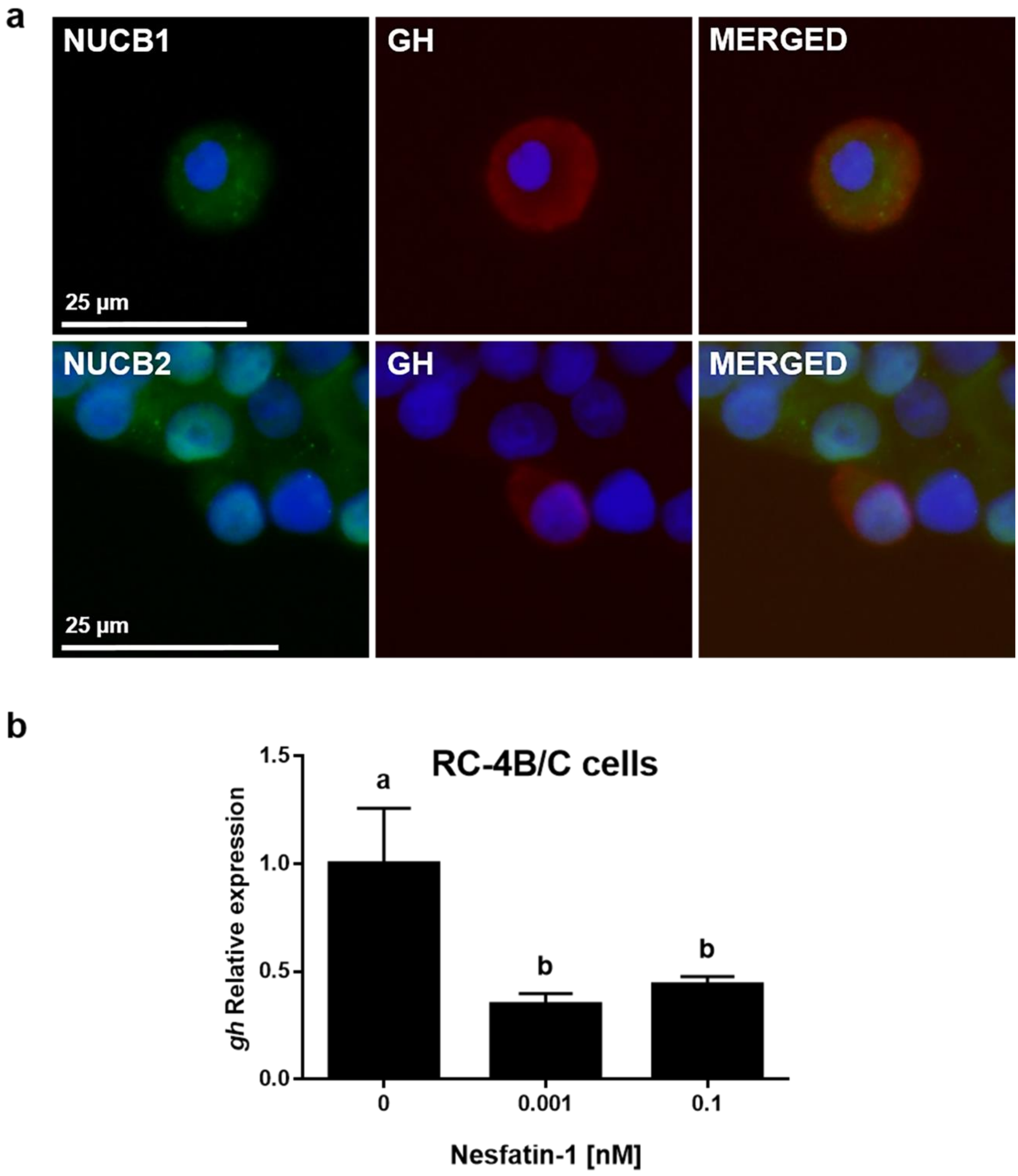

Fig. S2. (a) Colocalization of NUCB1 and NUCB2 with GH in RC-4-B/C cells. Representative images of immunofluorescence detection of NUCB1 (green), NUCB2 (green) and GH (red) in RC-4B/C cells. Cells were counterstained with DAPI (blue) and the images were acquired at 40X magnification. (**b**) **Effects of nesfatin-1 (NESF) incubation on *gh* gene expression in RC-4B/C cells**. Gene expression of *gh* after 1 h incubation with NESF in RC-4B/C cells. Data are shown as mean ± SEM (n = 12) relative to the reference genes *β-actin* and *rpl13*. Different letters indicate significant differences ($p < 0.05$) between the different concentrations detected by one-way ANOVA test followed by Tukey's multiple comparison test.

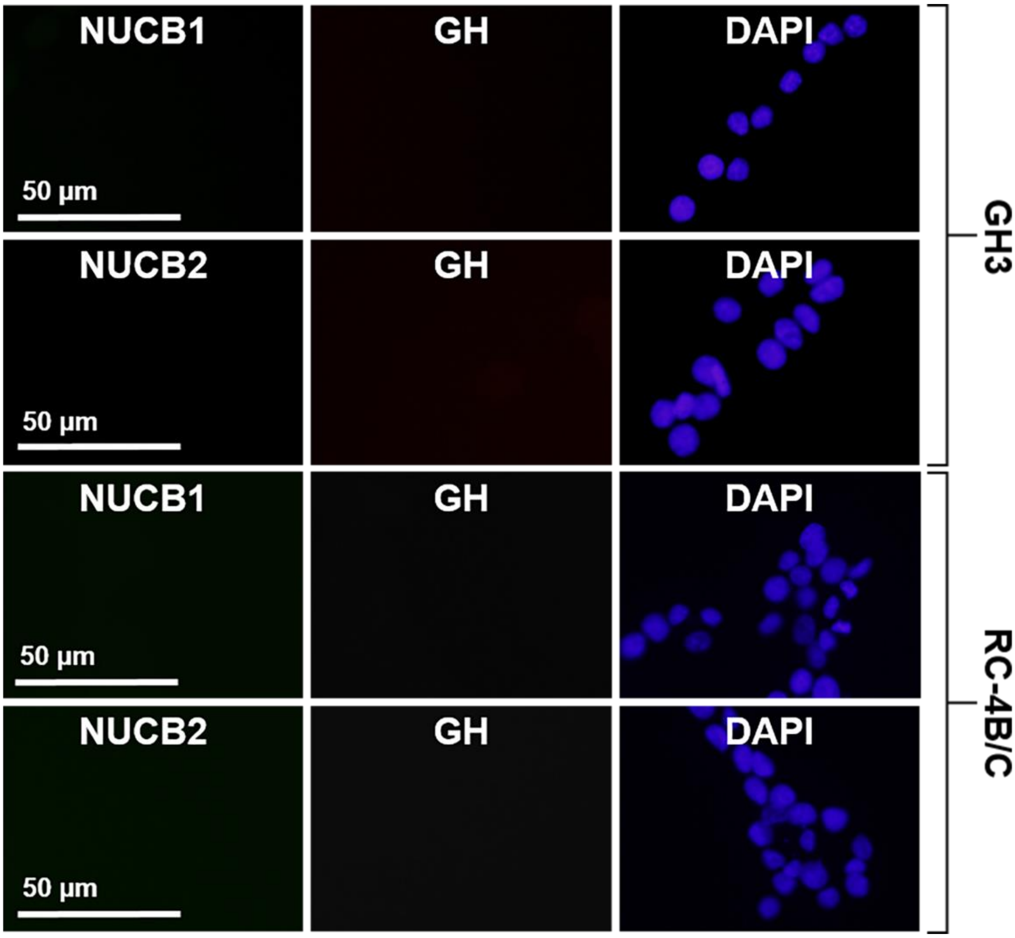

Fig. S3. No-primary antibody-negative controls of immunofluorescence for NUCB1, NUCB2 and GH in GH3 and RC-4B/C cells. Cells were counterstained with DAPI (blue) and the images were acquired at 40X magnification. No immunoreactivity was observed, confirming the specificity of the antibodies used.

Gene	Primer sequences (5'–3')	Tm ^{0}C	GenBank accession no.
Gapdh	**F**:CTACCCACGGCAAGTTCAAC<br>**R**:CCAGTAGACTCCACGACATAC	60	NM_01708
β-actin	**F**:CCCATCTATGAGGGTTACGC<br>**R**:TTTAATGTCACGCACGATTTC	60	NM_031144.3
Rpl13	**F**:GGATCCCTCCACCCTATGACA<br>**R**:CTGGTACTTCCACCCGACCTC	60	NM_173340
Nucb1	**F**:TGCCAACGCTGAGGACATTA<br>**R**:GTCCACCTGCAAGTTAGGCT	60	NM_053463.1
Nucb2	**F**:CCAGACACGGGACTTTATTATG<br>**R**:CCGCTCCTTATCTCCTCTATGT	60	NM_021663.2
Gh	**F**:TGGGCAGATCCTCAAGCAAA<br>**R**:ACACCAGTGTGTGCCTAGAAA	60	BC166872.1
Pit-1	**F**:GGAGAGCACAGCAAACCTTC<br>**R**:CGTTTTTCTCTCTGCCTTCG	60	L01507.1

Table S1. Forward (F) and reverse (R) primer sequences used for the qPCR analysis. qPCR conditions consisted of an initial activation step at 95 ^{0}C for 2 min, 40 cycles of 5 s. at 95 ^{0}C, 25 s. at 60 ^{0}C followed by a melting curve analysis through a temperature gradient of 65 to 95 ^{0}C with a 0.5 ^{0}C increase every 5 s.

Gels. Full-length agarose gels used to prepare **Figure 1a**.

Gene expression of *nucb1*, *nucb2* and *β-actin* in (a) <u>GH3</u>, and (b) <u>RC-4B/C</u> cells.
Each PCR product was separated by 1.5% agarose gel electrophoresis and visualized using RED Safe Nucleic Acid Staining Solution (cat no. 21141, FROGGABIO, Canada) in a ChemiDoc MP Imaging System (BIO-RAD, Canada). **NTC**: No Template Control. **RTC**: No Reverse Transcriptase Control. **PCRC**: PCR Control (No Template Control in the PCR).
Bands shown in **Fig. 1a** are boxed, and the molecular markers presented.

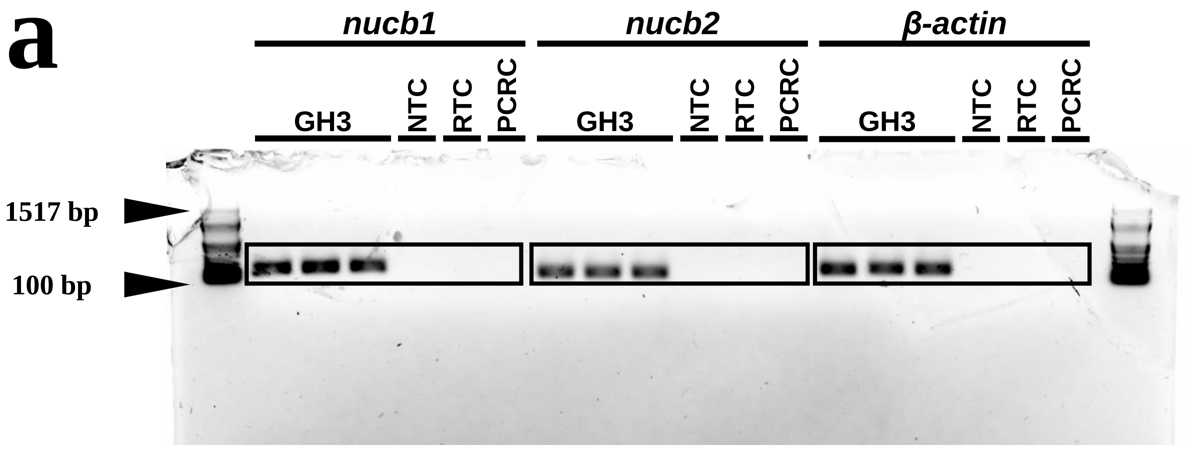

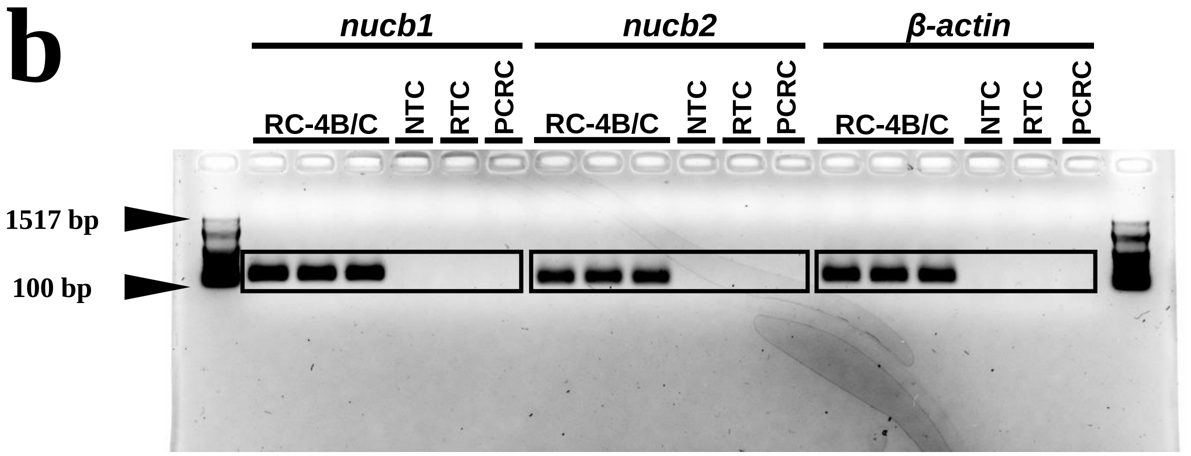

Blots. Full-length Western blot gels used to prepare the figures. Representative immunoreactive bands of the different experiments:

(a) NUCB1, NUCB2 and β-actin immunoreactivity in GH3 cells.
Bands shown in **Fig. 1b** are boxed, and the molecular markers presented.

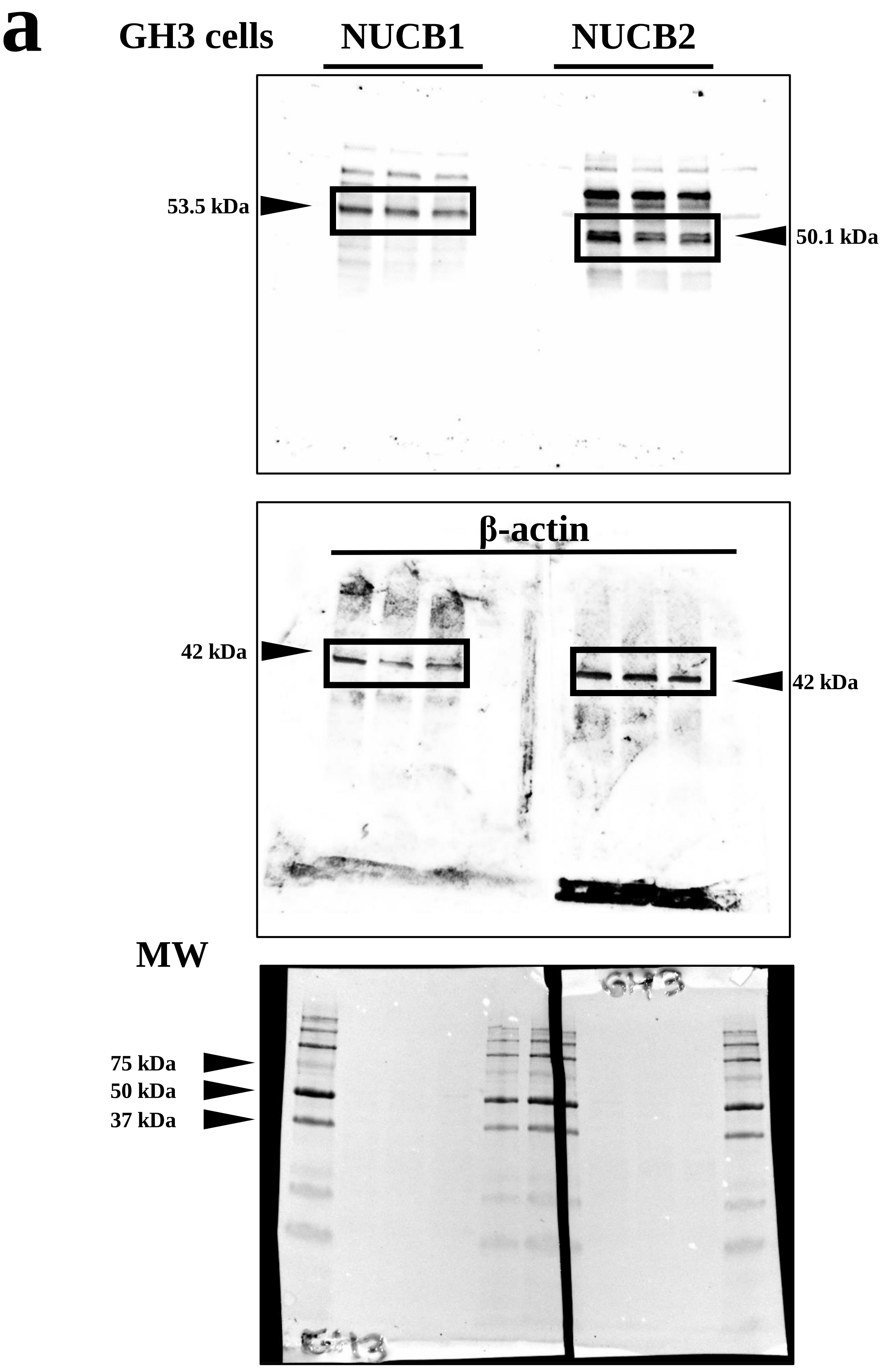

Blots. Full-length Western blot gels used to prepare the figures. Representative immunoreactive bands of the different experiments:

(b) NUCB1, NUCB2 and β-actin immunoreactivity in RC-4B/C cells.
Bands shown in **Fig. 1b** are boxed, and the molecular markers presented.

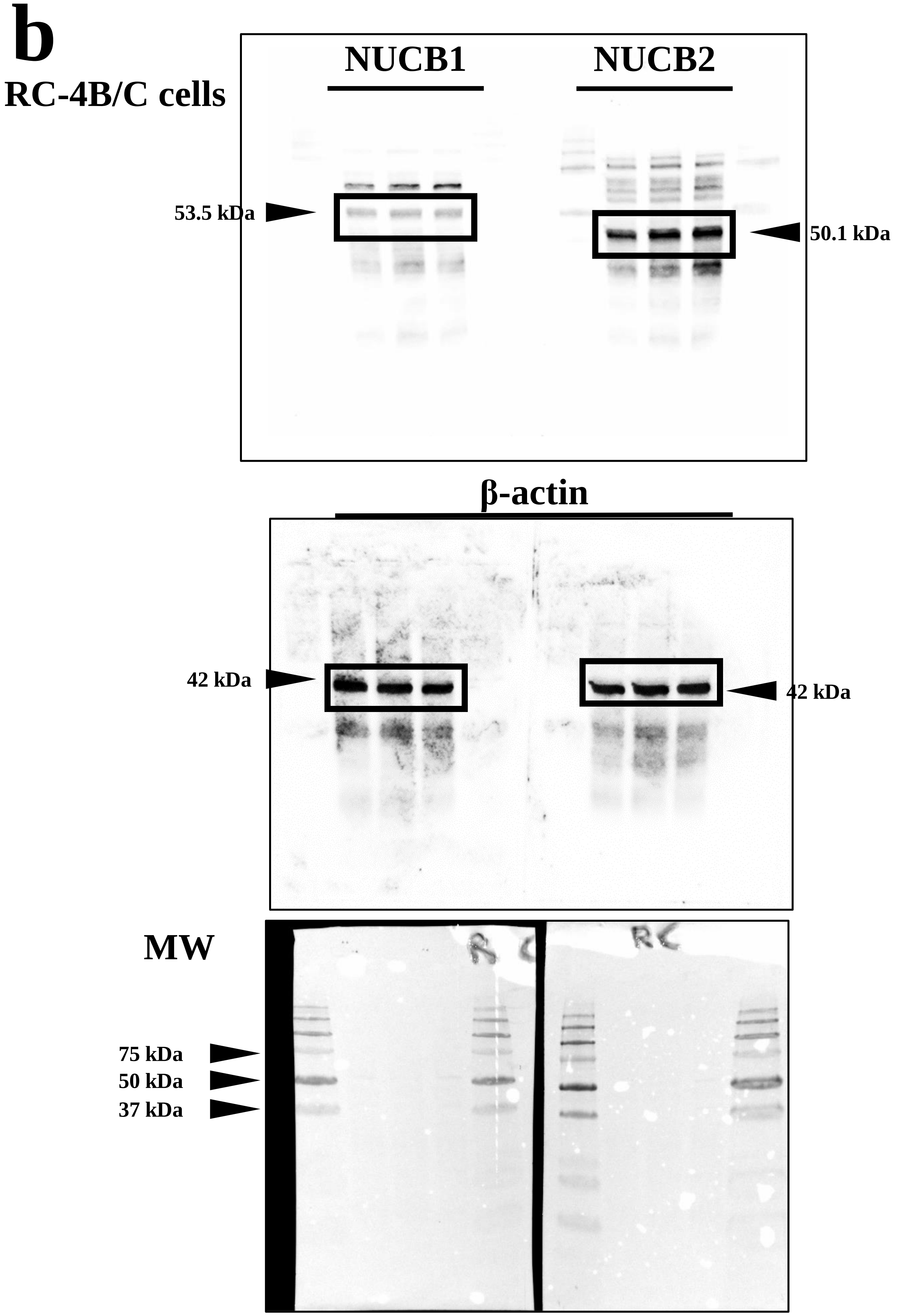

Blots. Full-length Western blot gels used to prepare the figures. Representative immunoreactive bands of the different experiments:

(c) GH and β-actin immunoreactivity in GH3 cells after 1h incubation with nesfatin-1 (NESF).
Bands shown in **Fig. 4a** are boxed, and the molecular markers presented.

c

NESF 1h

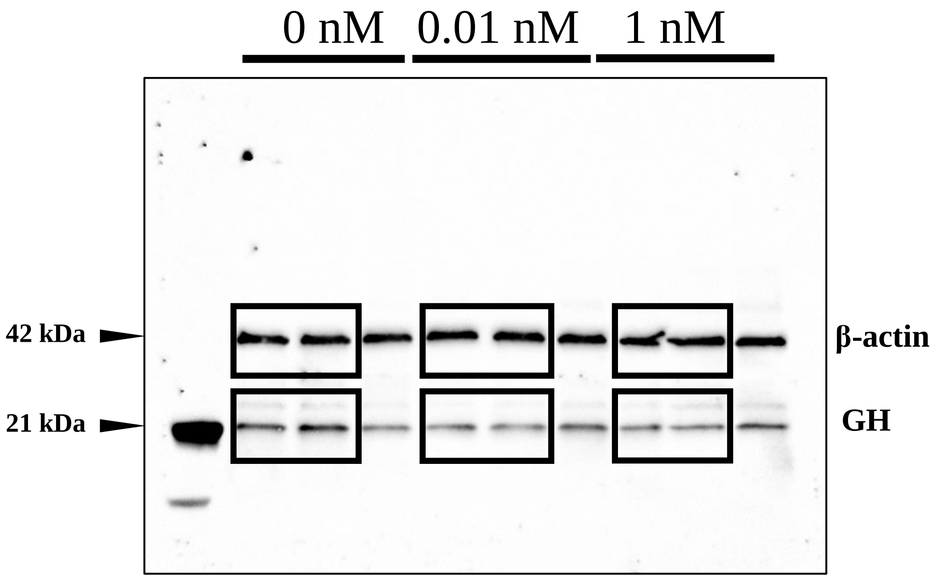

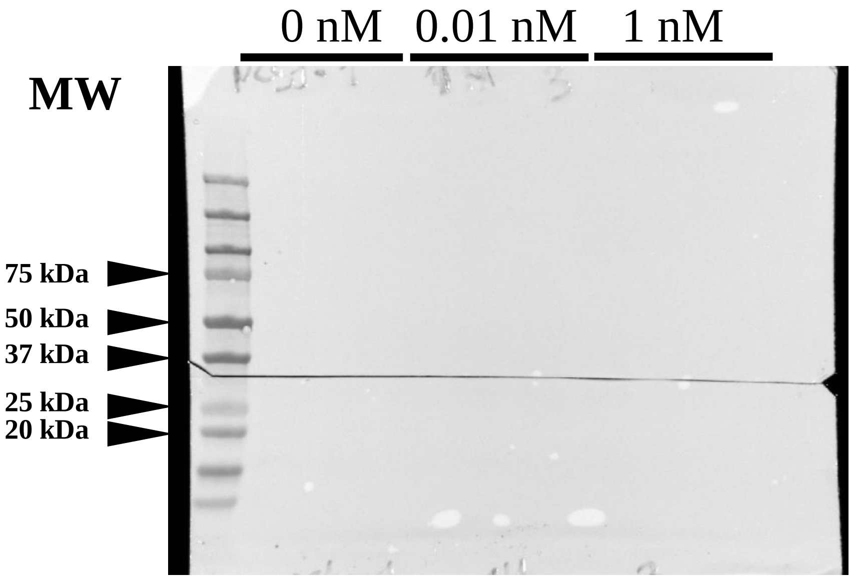

Blots. Full-length Western blot gels used to prepare the figures. Representative immunoreactive bands of the different experiments:

(d) GH and β-actin immunoreactivity in GH3 cells after 6h incubation with nesfatin-1 (NESF).
Bands shown in **Fig. 4b** are boxed, and the molecular markers presented.

d

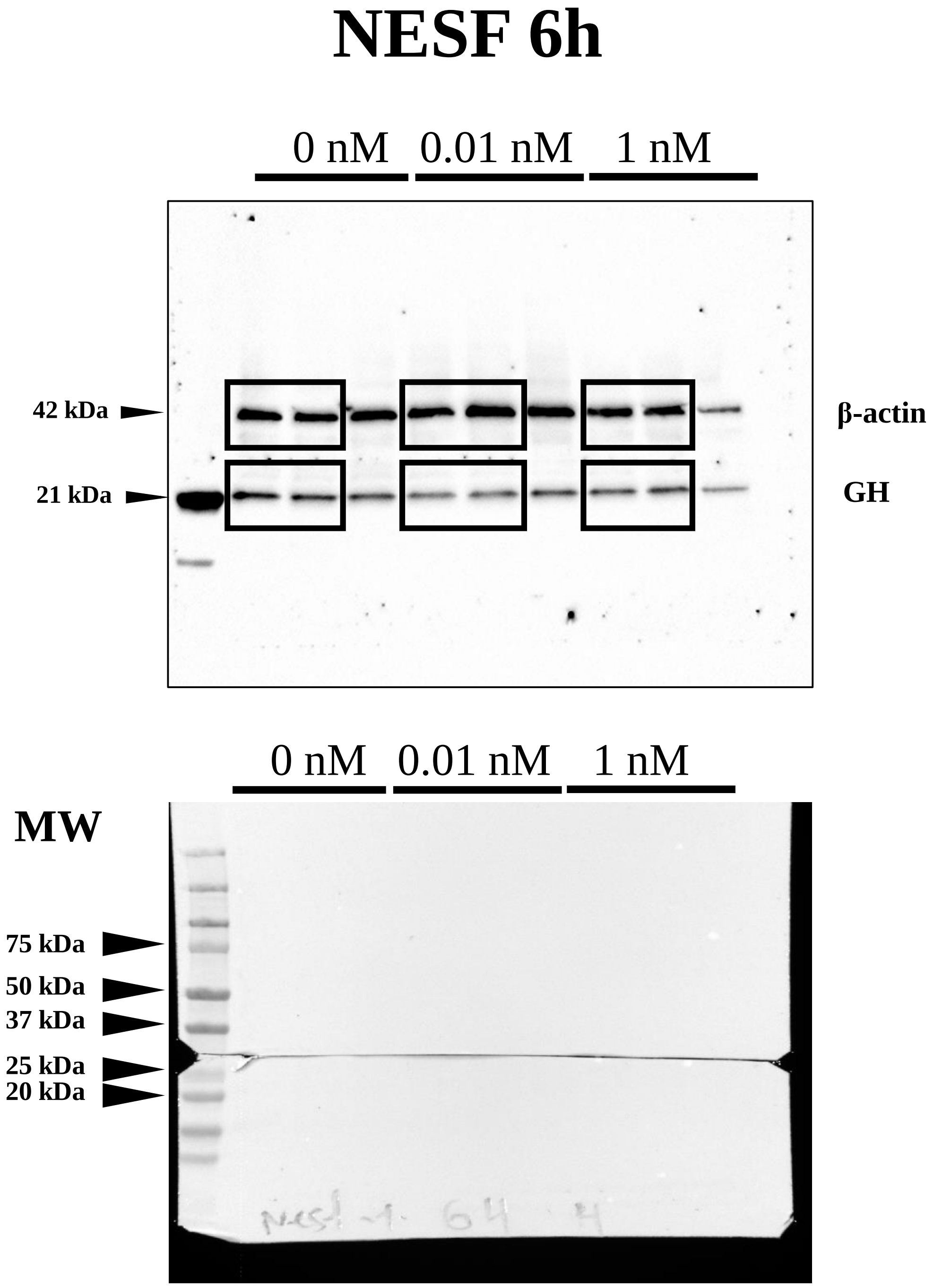

Blots. Full-length Western blot gels used to prepare the figures. Representative immunoreactive bands of the different experiments:

(e) GH and β-actin immunoreactivity in GH3 cells after 1h incubation with NLP.
Bands shown in **Fig. 4c** are boxed, and the molecular markers presented.

e

NLP 1h

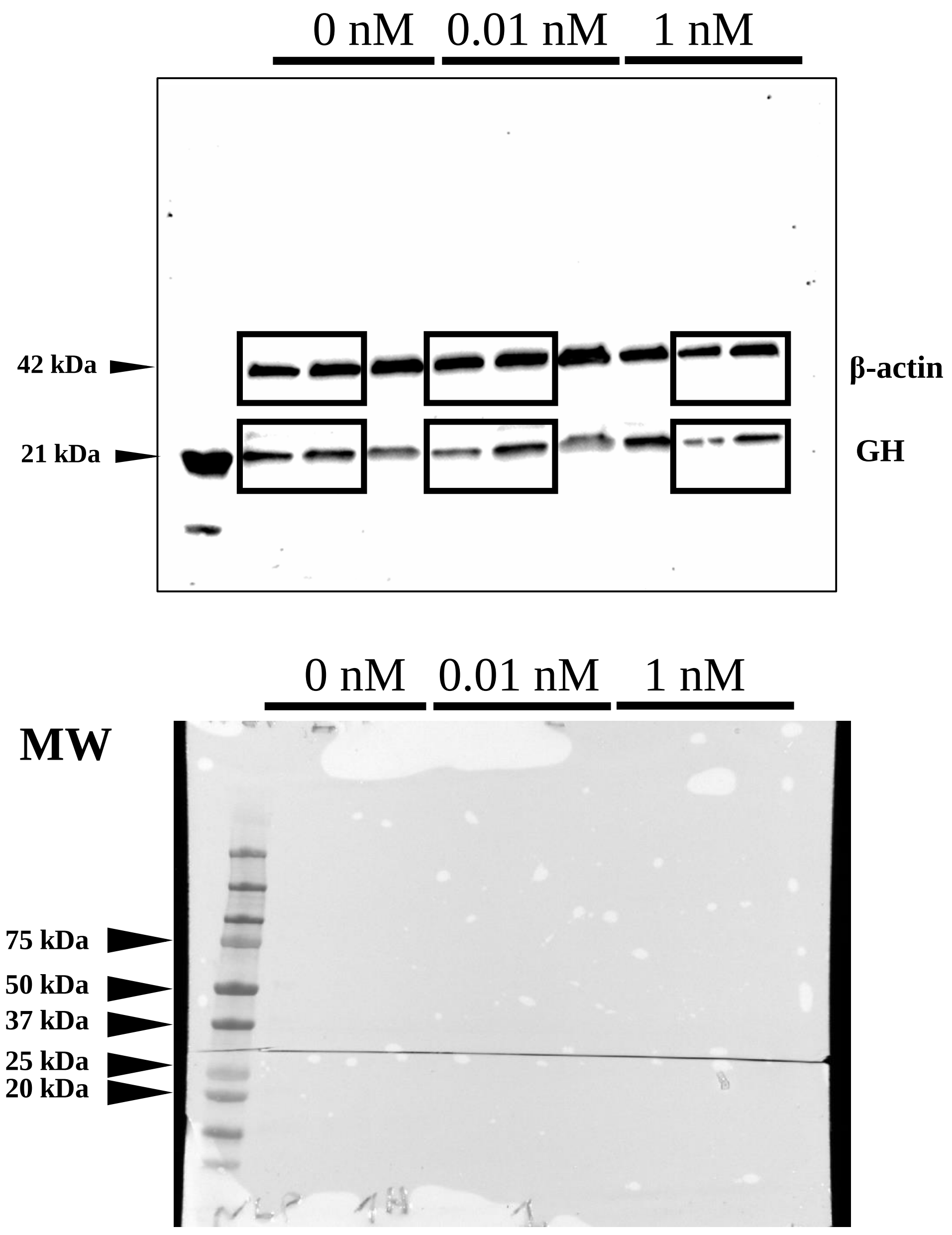

Blots. Full-length Western blot gels used to prepare the figures. Representative immunoreactive bands of the different experiments:

(f) GH and β-actin immunoreactivity in GH3 cells after 6h incubation with NLP.
Bands shown in **Fig. 4d** are boxed, and the molecular markers presented.

f

NLP 6h

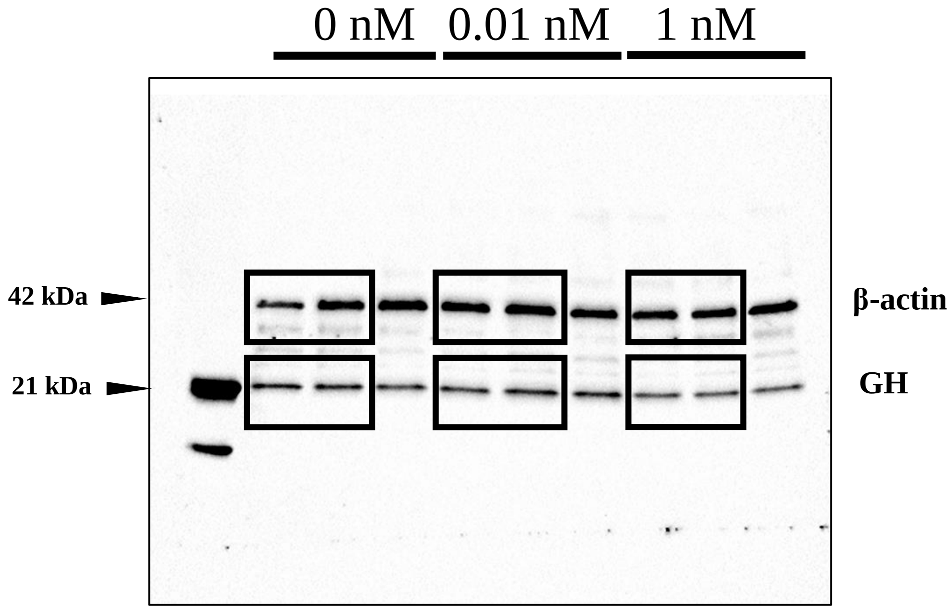

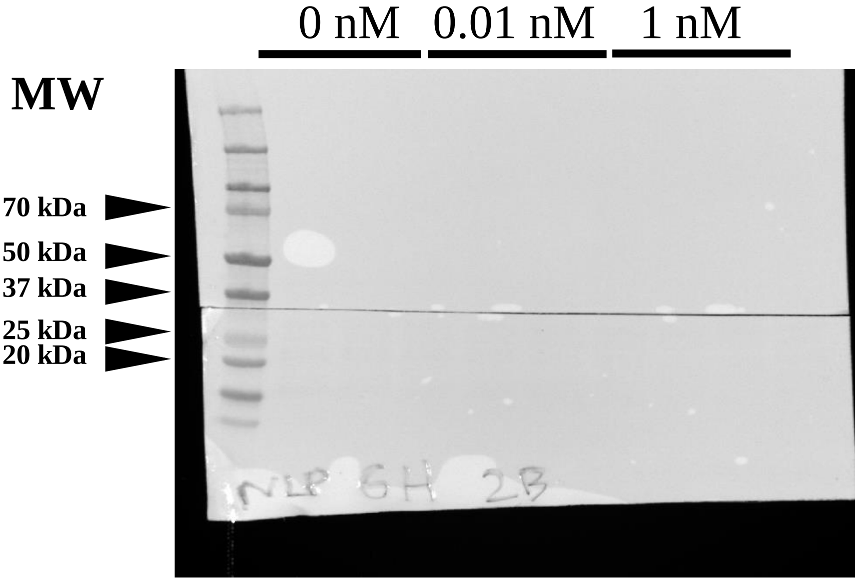

Blots. Full-length Western blot gels used to prepare the figures. Representative immunoreactive bands of the different experiments:

(g) P-CREB, T-CREB and β-actin immunoreactivity in the experiment with forskolin. Bands shown in **Fig. 6a** are boxed, and the molecular markers presented.

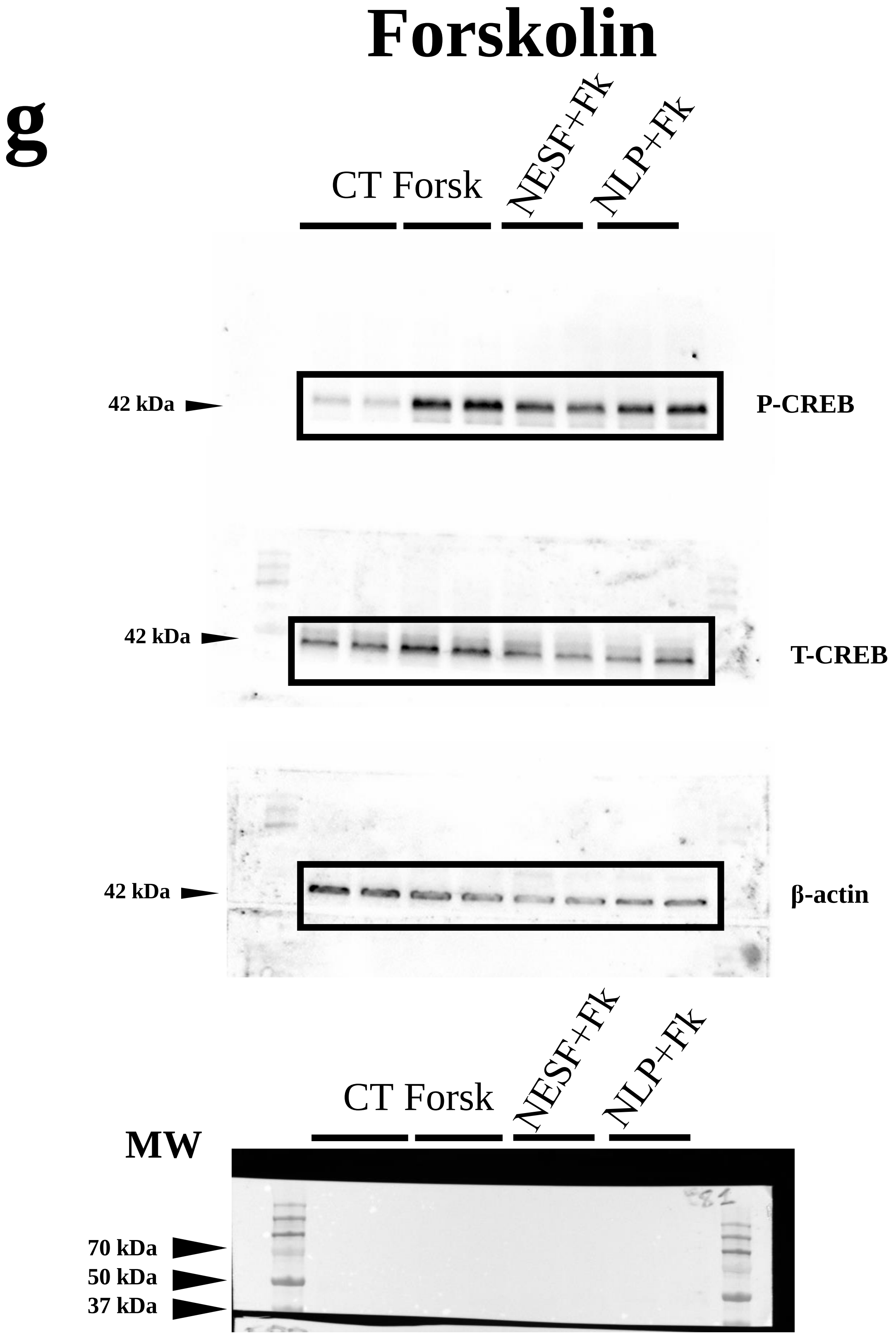

Blots. Full-length Western blot gels used to prepare the figures. Representative immunoreactive bands of the different experiments:

(h) P-CREB, T-CREB and β-actin immunoreactivity in the experiment with CPT. Bands shown in **Fig. 6b** are boxed, and the molecular markers presented.

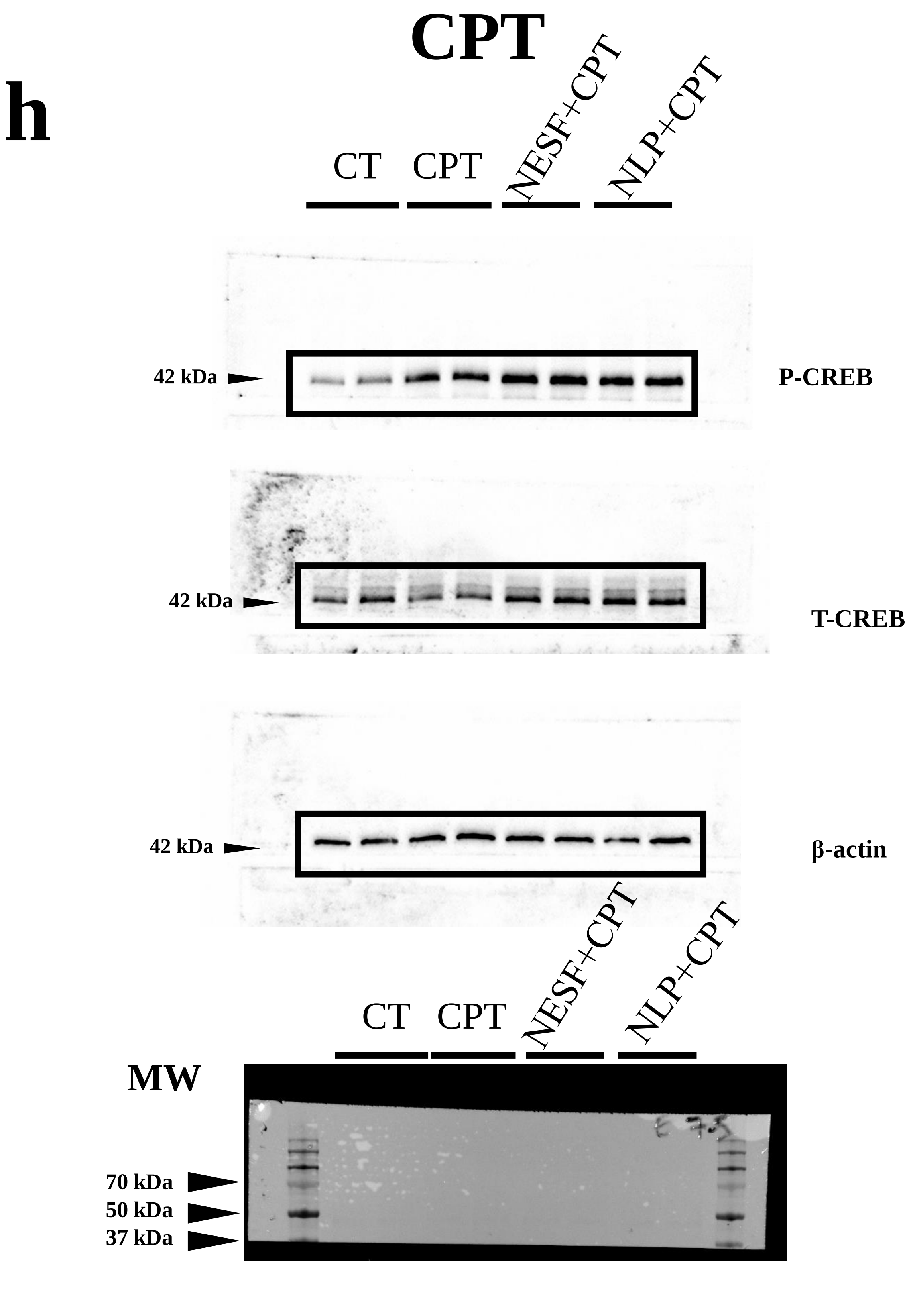

Blots. Full-length Western blot gels used to prepare the figures. Representative immunoreactive bands of the different experiments:

(i) P-CREB, T-CREB and β-actin immunoreactivity in the experiment with GRL. Bands shown in **Fig. 6c** are boxed, and the molecular markers presented.

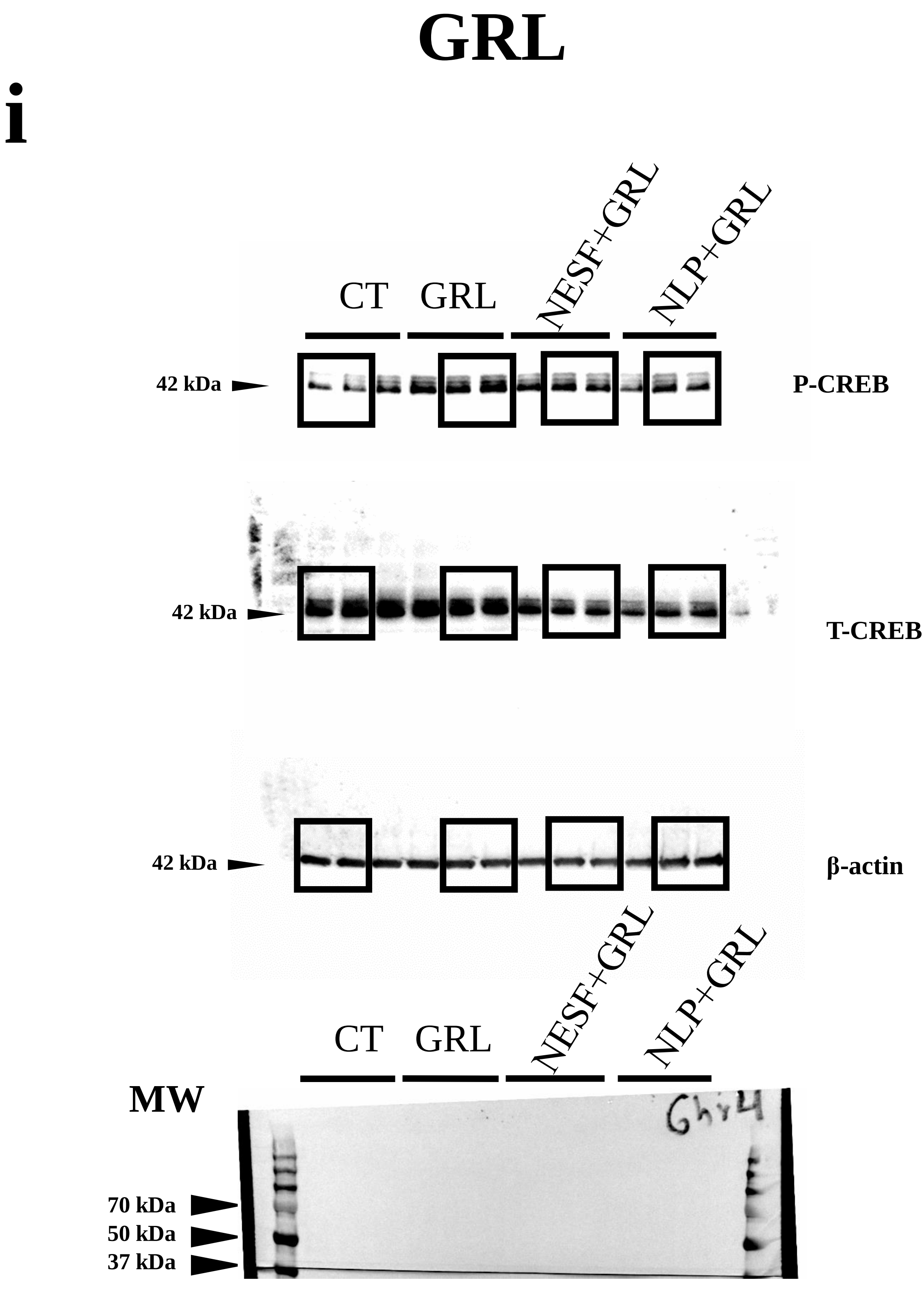

Supplement: Supplementary file 1 — Supplementary Information. [file 41598_2020_73840_MOESM1_ESM.pdf]
